# Minimally Invasive Fetoscopic Resection of Life-Threatening Amniotic Band Constrictions in a Human Fetus at 22 + 2 Weeks of Gestation Complicated by Subtotal Chorioamniotic Separation and Partial Placental Abruption

**DOI:** 10.3390/children12010020

**Published:** 2024-12-26

**Authors:** Nadja Riehle, Oliver Nowak, Leila Messroghli, Samantha Wakerlin, Thomas Schaible, Thomas Kohl

**Affiliations:** 1 German Center for Fetal Surgery & Minimally Invasive Therapy (DZFT), Theodor-Kutzer-Ufer 1-3, 68167 Mannheim, Germany; nadja.riehle@umm.de; 2 Department of Obstetrics & Gynaecology, University Medical Center Mannheim, Theodor-Kutzer-Ufer 1-3, 68167 Mannheim, Germany; oliver.nowak@umm.de; 3 Department of Anaesthesiology, University Medical Center Mannheim, Theodor-Kutzer-Ufer 1-3, 68167 Mannheim, Germany; leila.messroghli@umm.de; 4 Department of Surgery, University of California, San Francisco, CA 94158, USA; samantha.wakerlin@ucsf.edu; 5 Department of Neonatology, University Medical Center Mannheim, Theodor-Kutzer-Ufer 1-3, 68167 Mannheim, Germany; thomas.schaible@umm.de

**Keywords:** fetal surgery, fetal intervention, fetoscopy, amniotic band syndrome, placental abruption, chorioamniotic membrane separation, outcome

## Abstract

Amniotic band syndrome is a constrictive phenomenon in fetal development that can provoke limb autoamputation, malformation, trunk division, and umbilical cord strangulation. The latter two complications will ultimately lead to fetal demise if left untreated. If detected early enough, select cases may benefit from prenatal resection of the amniotic bands, thus preventing amputation and fetal death. Yet, especially in the presence of complete chorioamniotic separation, these procedures are rare, technically difficult, and not without significant risk. Objectives: The purpose of this report is to present the surgical technique and outcome of a challenging percutaneous fetoscopic intervention in a human fetus with amniotic band constrictions of a fetal thigh, retroplacental hematoma, partial placental abruption, subtotal chorioamniotic separation, and multiple amniotic bands encircling the umbilical cord. Methods: Minimally invasive, fetoscopic surgery to salvage the fetal life and lower leg was performed at 22 + 2 weeks of gestation under general maternofetal anesthesia. Results: Total resection of all amniotic bands was achieved, notwithstanding the aforementioned challenges. No surgical complications were observed. Despite preterm delivery at 25 + 4 weeks of gestation, the postnatal experience for the infant was favorable and uncomplicated as it furthermore benefitted from neonatal intensive care. At almost three years of age, the child remains healthy and demonstrates normal function of the formerly constricted leg. Conclusions: Our case shows that the combination of tested percutaneous fetoscopic techniques, high-risk obstetrics, and modern neonatology can overcome multiple obstacles in order to save a fetal patient stuck in a near-hopeless situation.

## 1. Introduction

Amniotic band syndrome is a rare condition that occurs during fetal development. This syndrome may result in a myriad of congenital malformations secondary to the entrapment of fetal parts by fibrous bands within the amniotic sac. It occurs with an incidence of approximately 1 in 2000 to 1 in 5000 pregnancies [1]. Amniotic band syndrome has been described for centuries, but the pathogenesis still remains elusive [1,2,3]. There has been much speculation regarding band formation and whether it occurs as a result of endogenous or exogenous factors. According to the endogenous theory, maldevelopment in the embryonic period leads to the disruption of embryonic vessels, which interferes with the normal development of fetal structures. As a result, necrosis of the body wall may occur, leading to the formation of these fibrous remnants [2]. The alternative exogenous theory suggests that mechanical trauma arising from accidental injury or iatrogenic intrauterine injury secondary to invasive procedures such as amniocentesis, fetoscopy, and open fetal surgery leads to band formation [3].

These fibrous bands are steadfast and lack elasticity, which makes them both constrictive and compromising to fetal growth. Amniotic bands can occur in isolation or in multiples, thus making the spectrum of complications diverse. When a band forms around a fetal limb, the fixed structure can cause acrosyndactyly or amputate the entire extremity. Complete chorioamniotic membrane separation, associated with an increasing number of amniotic bands, has also been observed. Amniotic bands encircling the neck, head, or trunk have also been the cause of gruesome craniofacial malformations and trunk divisions leading to fetal death [4,5]. When these structures entrap the umbilical cord, strangulation is inevitable and, if left untreated, will cause fetal demise as well [6].

Clinical management for amniotic band syndrome is determined on a case-by-case basis by gestational age at detection, expected severity, the presence or absence of umbilical cord strangulations, and maternal barriers to therapeutic intervention. Few prognostic indicators have been suggested that may aid in guiding the clinical management of amniotic band syndrome. Doppler ultrasound assessments of venous and arterial blood flow distal to a limb constriction can help in differentiating cases that could benefit from fetal surgery from those where the surgery would nevertheless be followed by limb loss or other irreversible injuries. [7]. The presence of arterial and venous blood flow, as well as active movements distal to the constriction, indicates that the entangled fetal part can still be salvaged by fetal intervention. In contrast, poor arterial flow alone and a lack of movement indicate loss of the affected limb region.

Nevertheless, more careful advocates of fetal surgery have suggested that—given their potential risks—such procedures should not be performed for limb salvage alone but be offered exclusively to life-threatening cases [8]. Complying with this recommendation, it has been our policy to consider and offer prenatal band removal in those cases with detectable bands encircling the umbilical cord. We have learned from previous untreated cases with cord constrictions that umbilical cord perfusion deteriorates quickly, as fetal death ensues in a period of days to weeks.

Yet, apart from identifying a fetus that may benefit from amniotic band removal, the procedure needs to be technically possible. Entering the amniotic sac alone has associated risks in fetoscopy, and the presence of chorioamniotic membrane separation adds yet another layer of complexity to the task. Chorioamniotic membrane separation has been identified as an additional risk factor for promoting premature rupture of membranes and preterm delivery [9]. The only documented fully percutaneous fetoscopic surgery of amniotic band syndrome in the presence of total chorioamniotic membrane separation with favorable outcomes in the literature was performed at our center in 2011 [10].

The purpose of this paper is to report an even more challenging intervention in a singleton fetus with amniotic band syndrome and subtotal chorioamniotic membrane separation, with the additional elements of partial placental abruption and intraamniotic hemorrhage. We present the surgical technique, clinical management, and outcome of this complicated case that endangered both mother and the fetus. We aim to highlight what can be achieved by combining percutaneous, minimally invasive, bimanual fetoscopic surgery with modern obstetrics and neonatology, accompanied by a good portion of luck on Christmas Eve.

## 2. Case

**Figure 1:**
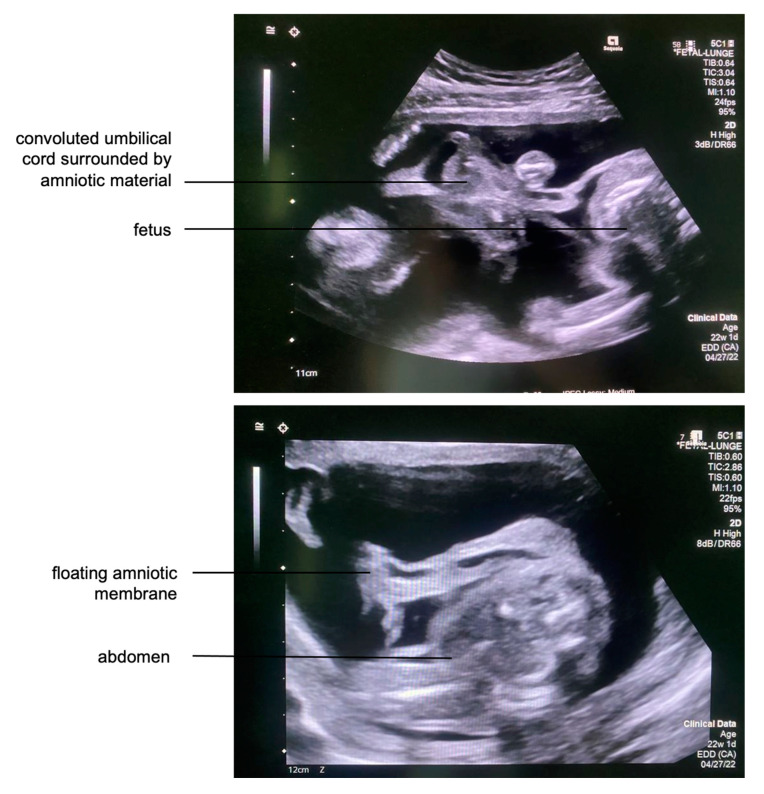
**Top***:* Transabdominal fetal ultrasound showing multiple strands of membranous material encircling and partially constricting the umbilical cord, indicative of a life-threatening state for the fetus. **Bottom***:* Due to subtotal chorioamniotic membrane separation, the subtotally disrupted amniotic membrane floated freely next to the fetus.

A 31-year-old pregnant woman (gravida 2, para 1) was referred to our center at 22 + 1 weeks of gestation after amniotic band syndrome had been detected in a previous routine ultrasound examination. All other screening examinations during her pregnancy were unremarkable. However, she did report vaginal bleeding in the first trimester. A maternal transabdominal fetal ultrasound revealed retroplacental hematoma, partial placental abruption, and subtotal chorioamniotic membrane separation, consistent with her history of vaginal bleeding in early pregnancy. Almost the entire amniotic membrane had separated from the chorion and floated freely in the amniotic fluid. The chorionic membrane seemed to be intact despite its separation from the amnion, as the amniotic fluid volume was normal and no vaginal fluid loss was seen. Multiple bands originating from the detached freely floating amniotic material and adhering to the fetus and its umbilical cord were observed (Figure 1).

**Figure 2:**
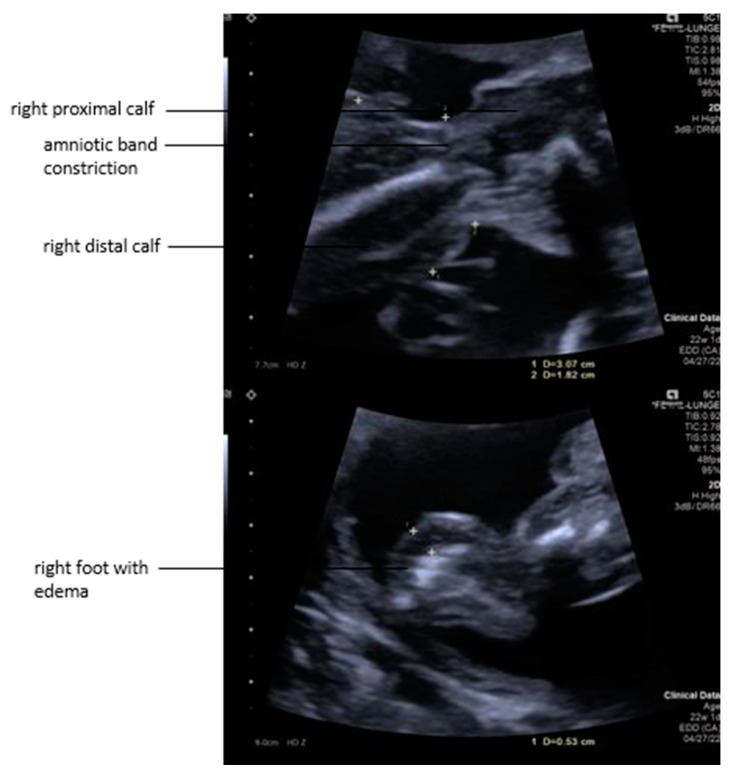
**Top***:* The fetal right calf was strangulated by an amniotic band with distal limb edema due to the constriction. **Bottom***:* The right foot became severely enlarged from the development of edema.

Several loops of the umbilical cord were entangled in amniotic tissue and parts of the abrupted placenta. Due to localized entrapment of umbilical cord slings and increased umbilical venous blood flow velocities, a constriction of the umbilical cord was suspected. Strangulation of the right calf was also noted, with associated edema extending down to the foot (Figure 2). A Doppler ultrasound assessment of the right leg indicated that the fetus might benefit from amniotic band removal as it showed decreased venous blood flow distal to the constriction, heralding the risk of limb loss without prompt intervention. Furthermore, the left leg protruded through the abrupted placental segment.

In order to alleviate the life-threatening cord constriction and to prevent amputation of the right lower leg, surgical treatment was offered to the parents. After receiving an explanation of the risks of severe maternal bleeding requiring immediate delivery, amniotic fluid leakage, premature rupture of the membranes, chorioamnionitis, or premature delivery by continuing the pregnancy—regardless of choosing the intervention or not—the mother gave consent to the intervention. The procedure was offered as one comprehensive and potentially life-saving treatment attempt. As such, no approval by our local ethics committee was required according to German law. Apart from this deviation, the procedure was performed according to the guidelines of the Declaration of Helsinki (https://www.wma.net/policies-post/wma-declaration-of-helsinki/, accessed on 7 December 2024). No experimental surgical steps were required for the intervention as all surgical techniques applied had been in use with low maternal and fetal risks for over 20 years at our center.

As adequate pain management is required to have the best technical success and overall outcome in percutaneous, fetoscopic procedures, the intervention was performed under general maternofetal anesthesia [11,12]. A maternal systolic blood pressure of 120 mmHg was maintained intraoperatively to provide a hemodynamically stable environment for the fetus. Doppler ultrasound examinations of the fetoplacental circulation and fetal heart rate were routinely monitored following the induction of anesthesia and throughout the surgery. Additional precautions were taken to ensure that an emergency Cesarean section could be performed at any stage in the operation if necessary.

Our minimally invasive, fetoscopic approach required the insertion of three 11-Fr sheaths, each with an internal diameter of 5 mm, to allow for a 3.5 mm 30-degree rod-lens fetoscope, a 3 mm endoscopic grasper, and various 3 mm endoscopic scissors to be inserted and utilized. The trocars were placed into the amniotic cavity via the Seldinger approach under continuous ultrasound guidance. Amnioinfusion with crystalloid solution was not necessary as no amniotic fluid leakage was observed, and the amniotic fluid levels were sufficient for the procedure to continue.

**Figure 3:**
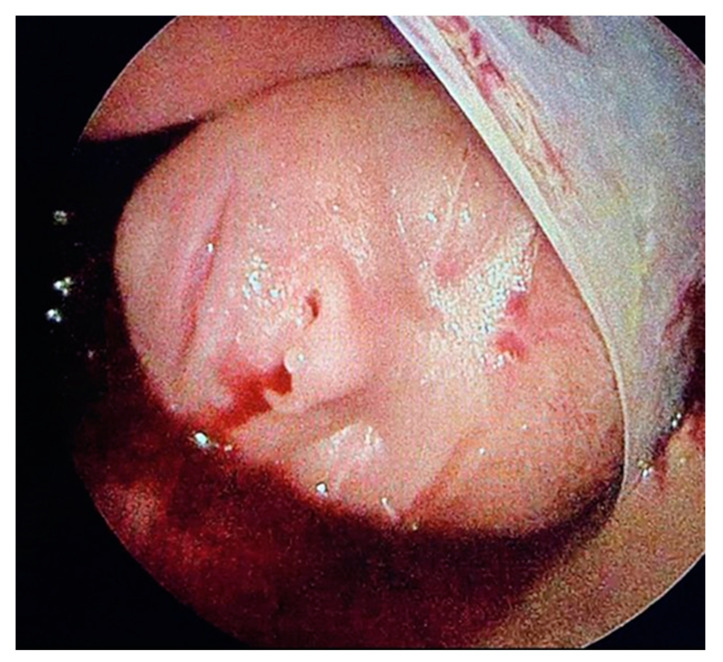
After entry to the amniotic cavity via three 11-Fr trocars, partial carbon dioxide insufflation (PACI) allowed for visualization of the fetal head, lying in a blood-tainted amniotic cavity and covered by the largely separated amniotic membrane.

**Figure 4:**
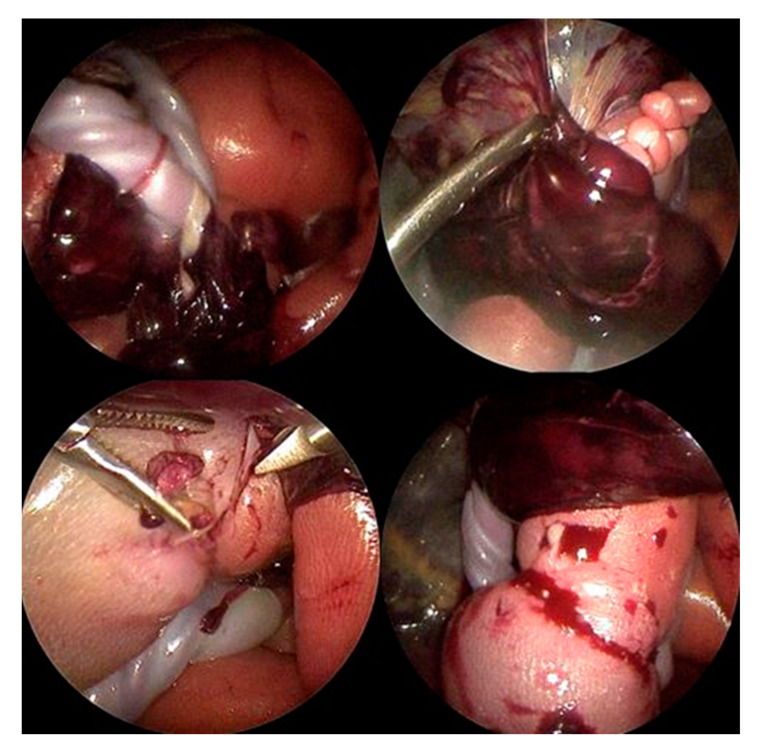
**Top left**: Multiple annular amniotic bands were entangling the umbilical cord and placental tissues. **Top right**: The fetal left foot was stuck within a part of the placenta, both restricting fetal movement and placing the mother and fetus at high risk of hemorrhage. **Bottom left**: A tight amniotic band encircling the right fetal calf caused severe strangulation, which would have led to partial limb autoamputation if left unresolved. **Bottom right**: After dissection of the right leg constriction due to the amniotic band, blood flow to the calf and foot improved immediately.

As the fetoscope was advanced into the amniotic cavity, fetoscopic inspection of the cavity and fetus was not possible as the amniotic fluid was severely blood-tainted and compromised visualization. Therefore, percutaneous partial amniotic carbon dioxide insufflation (PACI) was performed [13]. The mother was ventilated with target carbon dioxide levels within the physiologic range of 30–35 mmHg to prevent maternal and fetal respiratory acidosis during PACI. The insufflation pressure of the insufflator was increased in 2 mmHg increments until the release of carbon dioxide into the amniotic cavity was measurable. At this “opening pressure”, we removed the intrauterine fluid and exchanged its full volume with carbon dioxide. In this case, PACI was performed with an insufflation pressure of 8 mmHg; the flow rate was adjusted to 5 L/min. PACI permitted us to examine the whole amniotic cavity and the fetus (Figure 3 and Figure 4).

As already suspected in the ultrasound examination, the fetus was seen to lie in a blood-tainted amniotic cavity covered by amniotic material (Figure 3). As the left foot was protruding through a hole in the abrupted placenta, the fetus might have torn it off with a wrong movement, which, in the worst case, could have resulted in severe maternal hemorrhage. The umbilical cord was surrounded by multiple strings of the detached amniotic sac, and the proximal right calf was constricted by a tight, circumferential amniotic band (Figure 4).

Visualized by a 3.3 mm 30-degree rod-lens fetoscope, all amniotic bands, as well as the abrupted placental segment, were dissected using endoscopic scissors and graspers (Figure 4). Following dissection of the band that strangulated the proximal right calf, the extremity distal to the constriction quickly turned pink again, heralding promise for its preservation.

Subsequently, all strands of amniotic material that were twisting and entangling the umbilical cord were also dissected. After successful removal of all bands from both the fetus and its surroundings, to prevent new attachments, the insufflation was halted and the amniotic cavity was refilled with warmed, sterile crystalline solution. Following trocar removal, the maternal abdominal skin incisions were closed with single sutures.

The mother and the fetus tolerated the procedure fortuitously well. No complications were observed during surgery or over the remainder of gestation. After surgery, the patient stayed in the hospital for routine prophylactic tocolysis (Atosiban for 24 h; initial i.v. bolus dose of 6.75 mg, followed by intravenous infusion of 7.5 mg/mL at a rate of 24 mL/hour for 3 h, then a flow rate reduction to 8 mL/hour) and intravenous antibiotic (Clindamycin, 4 × 600 mg i.v. over 3 days). She was closely monitored in the gynecological ward for the early detection of complications like chorioamnionitis, bleeding, placental abruption, amniotic leakage, or premature contractions.

**Figure 5:**
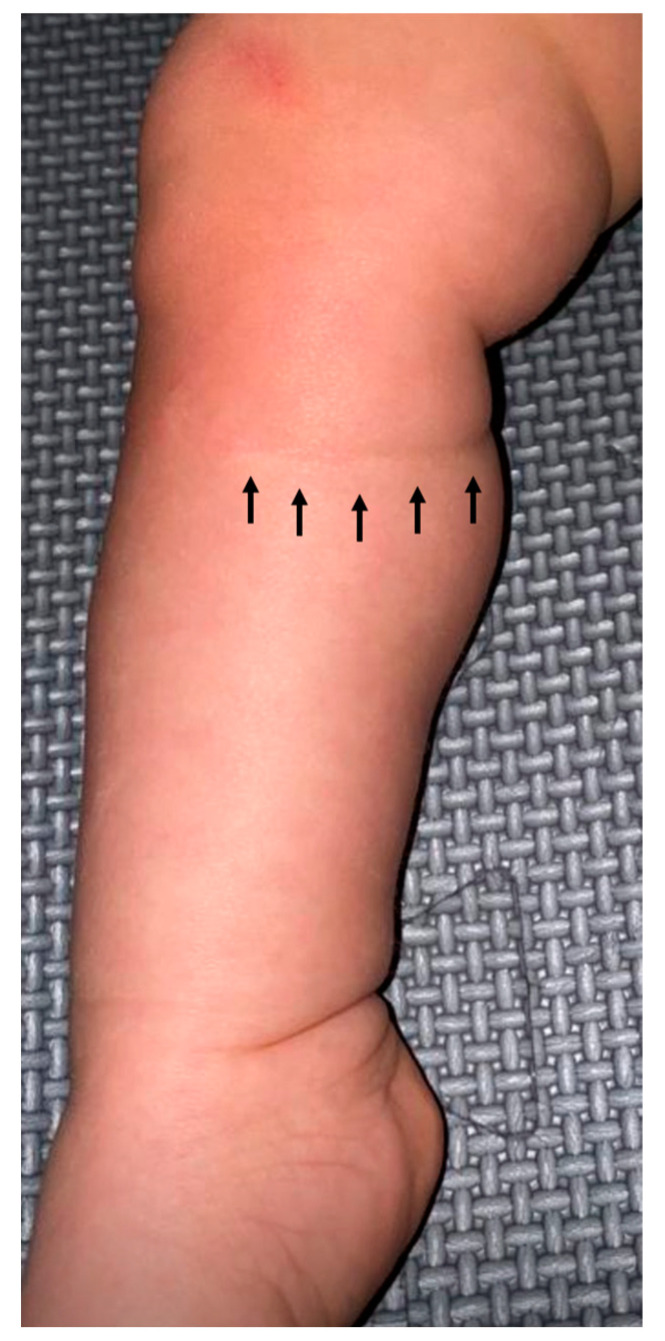
Postnatal aspect of the remaining band imprint (arrows) and the fortunately transient foot drop following prenatal fetoscopic removal of the tight amniotic band that had encircled the proximal right fetal calf. Foot position and function were noted to be normal by the end of the first year.

The pregnancy continued for 23 more days, bridging the most worrisome weeks for preterm delivery. When uterine contractions ultimately occurred at 25 + 4 weeks of gestation, the child was delivered by Caesarean section. At delivery, there were no band residuals on the neonate’s body. However, a circumferential imprint could be seen on the formerly constricted right calf (Figure 5). Movement of the right foot was temporarily impaired by a transient peroneal nerve paralysis. Over the course of the first year of life, supported by physical therapy, the foot drop resolved completely.

The tiny patient’s streak of luck continued, as all the most feared complications from preterm delivery at this very early age remained absent. After eleven weeks, the infant was discharged from hospital in good health. At about 3 years of age, the child remains well and exhibits normal development. He walks normally and has no sensory or motor impairments in his legs.

## 3. Discussion

This nearly impossible case demonstrates once again the life-saving technical possibilities of our range of fully percutaneous, minimally invasive, bimanual fetoscopic techniques that were initially developed by Kohl for fetal cardiac interventions. As multiple strands of membranous material encircled and partially constricted the umbilical cord in the mid-second trimester of gestation, the fetus was suffering from a truly life-threatening condition. In addition, one of its limbs would have undergone partial autoamputation without the intervention. Despite having been informed about the potentially life-threatening situation for herself, the mother decided not only against having the pregnancy terminated but for having her unborn child treated.

As the left foot of the fetus was stuck within the detached part of the placenta (Figure 4), the fetus could have fully abrupted the placenta with a wrong movement, which, in the worst case, would have resulted in severe hemorrhage for the mother. Therefore, the aim of this surgery was not only to improve the chance of fetal survival but also to mitigate the dangerous situation for the mother by pulling the left fetal foot out of the placenta completely. As all potential complications attributable to the procedure could have occurred during the natural, untreated disease course, there was justification to perform the operation in view of saving the fetal life.

Our standardized management before, during, and after the intervention ensured a high level of safety for the mother [12,13]. Apart from the surgical technicalities, the most important prerequisite to a safe and successful prenatal intervention is maternofetal general anesthesia according to a fetoscopy-specific protocol [12]. It was established at our center about two decades ago when it was observed that far lower dosages of maternal anesthetic were sufficient for achieving adequate uterine relaxation, maternofetal anesthesia, and the prevention of intraoperative uterine contractions.

The older protocols used during open fetal surgery—and some fetoscopic cases—recommended high dosages of volatile anesthetics for the same purposes, many of which caused maternal hypotension and impaired uteroplacental and fetoplacental blood flows. As the intraoperative treatment of maternal hypotension required high dosages of catecholamines, high volumes of crystalline fluids, and a battery of tocolytic agents, the iatrogenic fluid overload would often result in maternal pulmonary edema. In addition, the impaired uteroplacental and fetoplacental blood flows can explain the more frequent occurrence of fetal brain injury (leukomalacia) and intraoperative fetal demise [14,15]. As we learned that lower dosages of anesthetic drugs suffice for uterine relaxation and adequate pain management, these complications were not observed anymore beyond the learning curve of our management.

But what made the surgical implementation of the procedure possible in the first place was our approach of percutaneous partial amniotic carbon dioxide insufflation (PACI) and the bimanual approach. The amount of blood would have made it impossible to provide adequate fetoscopic visualization within a fluid medium. In addition, the complex spatial arrangements of the separated membranes, blood clots, and partially abrupted placenta, as well as the distribution and sheer number of amniotic strands, would have precluded the use of a single- or two-trocar fetoscopic approach [16].

The first fetoscopic procedures for releasing constrictive amniotic bands were described by Quintero and colleagues in 1997 [17]. Both procedures were hampered by impaired visualization, and both had to be completed with ultrasound guidance. Already at that time, Bruner and Tulipan were the first to employ carbon dioxide insufflation of the amniotic cavity during their pioneering attempts at introducing hybrid fetoscopic surgery for spina bifida [18].

Following multiple animal studies in sheep, it was Kohl who developed the principles and practice of a safe, fully percutaneous partial amniotic insufflation approach, which he called PACI [13]. From 2002 onwards, at the DZFT, PACI has not only enabled minimally invasive fetal spina bifida surgeries but has been instrumental in other intrauterine procedures of early pregnancy, including fetal tumor resections, amniotic band removals, umbilical cord ligations, fetoscopic laser ablations for twin-to-twin transfusion syndrome, and fetal cardiac interventions.

In our experience, no maternal complications, maternal deaths, or fetal deaths could be attributed to the PACI technique, ever. The most feared complication would be gas embolism, which we have also never observed in our entire series of more than 300 cases and which also remained absent during one of the early pioneering cases of fetoscopic hybrid spina bifida surgery by Joseph Bruner and Noel Tulipan, even when intraoperative placental abruption occurred [18]. More recently, other groups have observed blood gas readings within safe limits in human fetuses exposed to PACI, further supporting its safe use during fetoscopic surgery [19,20].

Standing on the shoulders of the pioneers of hybrid fetoscopic surgery and PACI, Belfort and colleagues described a two-port fetoscopic hybrid procedure using carbon dioxide insufflation of the amniotic cavity to save a fetal foot from amputation at 19 weeks of gestation by a ruptured and partially separated amnion.

The authors confirmed the superior visibility that can be achieved in a gas-filled uterus. In contrast to our minimally invasive, fully percutaneous procedure, the authors chose the more invasive hybrid approach, employing maternal laparotomy and exteriorizing the uterus. Choosing the hybrid approach in a non-life-threatening limb constriction, the lack of additional bands around the umbilical cord, and the absence of partial placental abruption with marked hemorrhaging into the amniotic cavity all point to a technically less challenging situation [21].

The fully percutaneous bimanual surgical approach was initially developed and clinically introduced by Kohl and colleagues [11] for fetal cardiac interventions and has safely been employed for more than two decades during more than 300 fetal interventions. This approach and its management were shared at length in a recent article by Kohl in Children to make it globally available to other fetal interventionalists [13]. In our experience of several hundred procedures, the fully percutaneous fetoscopic approach, performed in a sterile operating room environment, employing general maternofetal anesthesia, carries little intraoperative risk for both mother and fetus due to its minimal invasiveness. Before commencing surgery in our case, in order to further improve the intraoperative safety of mother and child, we made all preparations required to immediately switch to an emergency delivery if needed.

Apart from demonstrating the technical feasibility of fetoscopic intervention and clinical management in this high-risk case, our intraoperative observations may provide hints on its pathogenesis. Since our patient reported vaginal bleeding in early pregnancy, and since the amniotic bands were seen to originate from the detached amniotic sac and, prior to the procedure, were associated with partial placental abruption, it seems most likely that an exogenous trauma—rather than an endogenous maldevelopment in the embryologic period of the child—led to this life-threatening condition.

## 4. Conclusions

This case shows that the combination of tested, bimanual, minimally invasive, fully percutaneous fetoscopic techniques, high-risk obstetrics, and modern neonatology—accompanied by a streak of luck—can overcome multiple obstacles in order to save the life of a fetal patient stuck in a near-hopeless situation.

## Data Availability

Inquiries regarding any technical details of the procedure can be made to the corresponding author due to data security.
